# New Insights into Connection of Nucleolar Functions and Cancer

**Published:** 2019-03

**Authors:** Moslem Bahadori

**Affiliations:** Emeritus Professor and Consultant Pathologist, NIRTLD, Masih Daneshvari Hospital, Shahid Beheshti University of Medical Sciences, Tehran, Iran.

**Keywords:** Nucleolus, Ribosome biogenesis, Cancer phenotype, Gene stability

## Abstract

The nucleolus is an intranuclear membrane-less organelle. It is involved in ribosome biogenesis and protein synthesis. When the demand for protein synthesis increases in cell growth and proliferation (e.g., tumors), the cell upregulates ribosome biogenesis. Changes in nucleolar size and number have been recognized as known features of many tumor types. Recent evidence suggests that overproduction of ribosome, decreased ribosome biogenesis, and quantitative and qualitative changes in the nucleolus function, may result in oncogenesis. Today, it is clear that the nucleolus is involved in processes other than ribosome biogenesis. Other functions of the nucleolus include detecting and responding to endogenous and exogenous stress, maintaining genome stability, and regulating cell cycle progression, telomere function, cellular senescence, gene expression, and chromatin structure. Alterations in many of these fundamental nucleolar processes may contribute to the formation of cancer cell phenotypes. This phenomenon suggests that normal nucleolar functions are a safeguard against the development of malignancies and have potential therapeutic effects, as reported in non-small-cell lung carcinoma and other malignancies.

## INTRODUCTION

The connection between the nucleolus and cancer has been a subject of research since the 19^th^ century. Giuseppe Pianese, an Italian pathologist, observed the nucleolar morphology of cancer cells and their changes in 1896. He reported that hypertrophy and irregular shape of nucleoli were characteristics of malignant cells (1). Since then, there have been controversies regarding nucleolar changes due to cancer and the causative nature of neoplastic transformation.

Traditionally, the nucleolus has been known as a coordinator of ribosomal biogenesis and protein production and may overreact during tumorigenesis. Recently, knowledge about ribosome biogenesis and non-ribosomal functions has increased. Multiple studies have clearly shown that the nucleolus has many other functions, either ribosomal or non-ribosomal, including a role in cancer development (2). In this study, we briefly discuss recent findings on nucleolar connection to neoplastic transformations. For this purpose, we searched Google Scholar, Scopus, and Medline databases and relied on our experience to briefly discuss the established activities of the nucleolus in cancer.

### Nucleolus

In eukaryotic cells, the nucleolus is an intranuclear membrane-less organelle. It is visible in the interphase of cell division via light microscopy. Among nuclear components, the nucleolus takes up to 25% of the nuclear volume. The nucleolus is made up of protein and ribonucleic acid (2–5). The nucleoli are not membrane-bound organelles. Under electron microscopy, the nucleolus is composed of three distinct regions. The pale-staining fibrillar center (FC) appears as a roundish structure of different sizes, surrounded by dense fibrillar components (DFC), which frequently constitute a rim intimately associated with FC. It is encased by an outer granular component (GC), composed of granules. GCs surround fibrillar structures and are also defined by their appropriate proteins or RNAs.

Ribosomal genes are active in the FC region and are closely associated with DFC. Therefore, FC plus DFC can be seen as functioning structural units of the nucleolus, producing rRNA molecules, which migrate to GC and undergo maturation (6, 7). The nucleolus disassembles during cell division in the interphase stage and then reassembles at the end of mitosis in the metaphase stage around a specific region, known as the nucleolar organizer region (NOR), which can be visualized as dark dots under light microscopy via silver staining (AgNOR). The nucleoli are formed around NOR on acrocentric chromosomes 13, 14, 15, 21, and 22. Several NORs may exist within a species, each residing on a separate chromosome. The nucleolus around each NOR contains clusters of approximately 300 ribosomal RNA gene repeats (rDNAs) (8, 9).

### Ribogenesis

Traditionally, the main function of the nucleolus is ribosome biogenesis, which is a complex molecular machine responsible for protein production. Its major function is translation of mRNA into a variety of proteins. This activity is regulated by many factors at different levels. It begins with the transcription of ribosomal RNA (rRNA) in the nucleolus through RNA polymerase I (RNA Pol1) activity, generating pre-rRNA. In turn, pre-RNA is activated, modified, and cleaved to form derivatives of rRNA; this process continues until mature rRNA is processed. Next, through transcription by RNA polymerase II (RNA Pol II), mature species assemble with RP and are then transcribed by RNA polymerase III (RNA Pol III). Finally, they are exported from the nucleus to the cytoplasm and increase cytoplasmic ribosomes. Accordingly, the produced ribosomes act as central players in mRNA translation into proteins (6, 7, 10, 11).

Because of the crucial role of the nucleolus in ribosome biogenesis, it can actively determine the metabolic condition of a cell. In morphology, the size of the nucleolus is positively correlated with rRNA synthesis, which is related to cell growth and metabolism. This nucleolar change can be observed by light microscopy after silver staining (AgNOR) (12, 13).

### New insights into nucleolar function

For a long time, ribosome biogenesis in protein synthesis was considered as the only function of the nucleolus. However, in the past two decades, the role of the nucleolus in cellular function has been greatly recognized. Researchers have reported that the nucleolus function is not simply ribosome production, and pathologists identified a subnuclear structure, responsible for changes in the nucleolus size and shape in cancer cells (14). Today, it is believed that the nucleolus is a key controller of many cellular processes, which are fundamental to normal cell homeostasis. In addition, its dysregulation can be the target of many human diseases. Previously, the nucleus was considered as the brain of the cell, while today, it is regarded as the brain of the nucleus (2, 4, 7, 14, 15).

High-throughput proteomics, genomics, and biochemical assays have shown that only 30% of nucleolar proteomes are involved in conventional ribosome biogenesis (16, 17). There are other roles for the nucleolus under physiological and pathological conditions. Many reports have indicated that nucleolar mechanisms control embryonic development, stem cell differentiation, and shuttling of regulatory molecules in the cell cycle and transcription factors. The nucleolus determines the cell lineage for the nucleoplasm and executes a differential program. The role of nucleolus in several processes, such as DNA damage sensing and repairing, maintenance of genome stability, and spatial organization, has been suggested.

Moreover, the nucleolus is involved in epigenetic regulation, control of the cell cycle, and stress response. It also contributes to cellular senescence, telomere function, chromatin structure, establishment of nuclear architecture, regulation of gene expression, and production of multiple ribonucleoproteins (18, 19). These newly discovered extranucleolar functions are termed as “non-canonical functions” to distinguish them from the traditional role of the nucleolus in ribosome synthesis. These non-canonical functions are the focus of today’s research.

New findings following the identification of nucleolar microRNAs (miRNA, snoRNA, and non-coding RNAs) have been topics of research for understanding the nucleolar capacity and function. It is known that miRNAs are non-coding RNAs with nearly 22 nucleotides, providing valuable information about the nucleolar function, especially in cancer involvement. Previously, it was believed that miRNAs can be only found in the nucleus, while today, researchers believe that they are the key players of the nucleolus in health and diseases, such as cancer (20–22).

There is emerging evidence on nucleolar functions in physiological activities, such as lifespan regulation, longevity, senescence, and aging in different models, involving C-elegans, drosophila, mice, and even humans. These models have been also used in studies on the pathological functions of the nucleolus. Reports indicate that many diseases, such as neurodegenerative brain diseases, cardiovascular dysfunction, viral infection, autoimmune disease, and cancer, are associated with nucleolar dysfunction (2, 4, 7).

## NUCLEOLUS IN CANCER

### Gene stability

The nucleolus promotes gene stability. The repetitive structure of rDNA, with its significant role in transcription, makes rDNA highly susceptible to genome instability and damage. The nucleolus reacts to these DNA damages by activating diverse signaling pathways. DNA damage response proteins (DDRP), which operate in the nucleolus, play a repairing role, as well. Upon DNA damage, a number of DDRPs accumulate in the nucleolus as an intranuclear body (INB), and the nucleolar cap provides a platform for recruiting specific factors, which sense and repair damage to gene stability of the nucleolus (23, 24).

### Nucleolar stress

When there are adverse growth conditions, metabolic deficiency, or oxidative stress, the nucleolus ribosomal RNA synthesis is downregulated through mechanisms involving polymerase transcription factors and epigenetic modification. This perturbation of nucleolar activity and integrity has been defined as nucleolar stress. Generally, this type of stress is very important in both health and disease (25).

### Connection of nucleolar function with cancer

The nucleolus connection with cancer can be viewed from two perspectives (intermingling may also happen):

- Changes in the nucleolus are a consequence of neoplastic transformation (1, 2).
- Functional nucleolar dysregulation by any reason is a risk factor for developing cancer (3, 4, 26).

The nucleolus, as a major contributor to ribosome production, is associated with cell cycle regulation in several subtle ways. One of these ways is the surveillance system. When rRNA synthesis or processing is impaired, the system reacts promptly, halting cell cycle progression. Conversely, the nucleolus acts as the first responder to stress signals related to growth factors. The cells must increase the protein synthetic capacity, in addition to overproduction of ribosomes to meet the increased demands of proteins during cell growth and proliferation; this process can change the nucleolar structure. Therefore, nucleolar size and number may be seen as hallmarks of tumorigenic activity.

The large size and increased count of the nucleolus are commonly recognized as the hallmarks of cancerous tumor cells; however, recent evidence suggests that this is not always true. In addition, decreased ribosome biogenesis, besides qualitative alterations in its production, can contribute to tumorigenic processes and cancer progression. As mentioned earlier, researchers have focused on nucleolar functions in processes that are clearly unrelated to ribosome biogenesis. These functions include sensing and responding to endogenous and exogenous stressors and DNA damage response, which are known as nucleolar functions in tumorigenesis (27, 28).

With respect to the traditional role of the nucleolus in ribosomal biogenesis and proliferative capacity, its contribution to cancer is well established. Many recent studies have shown that this intranuclear organelle plays a much more significant role in malignant transformations. The non-canonical function of the nucleolus is a key contributor to cellular proliferation and stress signaling. Recent findings show that nucleoli function as risk factors for cellular transformation in malignancies. Practically, nucleolar changes have been observed in all human cancers, but they seem to be highly variable. Changes are independent of the histogenesis of tumors and can be seen even within the same tumor (29, 30). Their functions in tumors are closely associated with the number of proliferating cells within the cancer tissue and rapidity of proliferation in cell cycle kinetic parameters. Increase of nucleolar functions in proliferating cells is related to the products of proto-oncogene and/or tumor suppressor genes that contribute to cell proliferation.

One of the nucleolus tumorigenic activities is to change proto-oncogenes and tumor suppressor genes. The C-Myc oncogene, which is necessary and sufficient for cell-cycle entry, is overexpressed in tumors. These genetic changes lead to neoplastic transformations and are of special importance. Other genes, involved in ribosome biogenesis, include retinoblastoma tumor suppressor protein (pRB) and p53 (31–33). The primary function of accelerated ribosome biogenesis in cancer has been proposed as the only factor resulting in increased proliferative growth, which is frequently associated with malignancy. Hepatocellular carcinoma following chronic HBV or HCV infection or inborn errors of metabolism is one of these malignancies.

Numerous studies suggest that non-ribosomal functions of the nucleolus, in particular controlling the activity of various critical tumor suppressors and oncogenes, significantly contribute to malignant transformations. Alteration and dysregulation of ribosome biogenesis, as a causative factor in cellular transformation, have been reported in many diseases. One of these diseases is an inherited syndrome, called dyskeratosis congenita (DKC), which is attributed to a mutation in *DKCI* gene. DKCI gene produces a nucleolar protein, called dyskerin, which is a member of small nucleolar ribonucleoloprotein (snoRNP) family, involved in RNA processing (34–36).

The nucleolus may become the central component of oncogenesis through the following pathways: growth and survival derangement, stress responses, DNA damage responses, senescence, telomeres, and genome regulation. The outcome may be sustaining proliferative signaling, emergence of growth suppressors/depressors, cell cycle damage, genome instability and mutation, enabling replicative immortality, activating invasion and metastasis, and resisting cell death (26). Nearly all cancers display large nucleoli and/or increased count. This can be visualized by light microscopy after AgNOR staining. In fact, nucleolar size in some cancers can be used as a predictive indicator of clinical outcomes. Generally, increase of the nucleolar size suggests poor prognosis. Nucleolar size has been also suggested as a measure of response to chemotherapeutic drugs (26). Evidence shows that increased rates of Pol I transcription in the nucleolus are correlated with poor prognosis; this finding suggests the use of cancer chemotherapy.

Alteration of regulatory proteins in the cell cycle is a common feature of most cancer types. The CDK-cyclinD/INK4/pRB/E2F pathway contributes to the regulation of G1/S transition. This change, which is commonly observed in cancers, is a topic of therapeutic research. Several studies have investigated various nucleolar proteins, such as pRB, Myc family, P53, ARF, and dyskerin (3, 26, 27). Substantial evidence shows that extra-ribosomal nuclear functions play major roles in cancer. Advances in proteomic analysis techniques for nucleolus proteins have demonstrated pluripotent activities. More than 4500 proteins have been identified in the nucleolar proteome database (NOPdb). Less than 50% of these proteins function in traditional ribosome biogenesis. The remaining nucleolar proteins are involved in a wide range of functions, including the regulation of tumor suppressor and proto-oncogene activities, cell cycle control, and gene stability (16, 37).

Accumulating evidence suggests that dysregulation of small nucleolar RNAs (snoRNAs) plays a role in the development of lung cancer. These molecules have been shown in tumor-initiating cells (TICs) of the lungs, responsible for tumor progression and recurrence. They cause malfunctions in tumor initiation and progression in the lungs. Researchers have identified many snoRNAs, with changes specific to TICs. Different scholars have proposed a list of snoRNAs. Reports show either their tumorigenic activity or their inhibition for therapeutic purposes. Contribution of snoRNAs to lung TICs provides potential biomarkers for predicting the outcome of non-small-cell lung cancer (NSCLC) (34). For example, snoRNA 42 is one of the important snoRNA biomarkers in identifying lung TICs, progressing to lung tumor. The expression level of other snoRNAs has been quantitatively presented via real-time polymerase chain reaction (PCR) assay. Many reports have shown that targeting various oncogenes and tumor suppressors and inhibition of snoRNAs are controlling factors in lung cancer ( 38–40).

### Anti-cancer functions

Increase of rDNA transcription has been shown to be a common feature of human cancer. It depends on nucleolar proteins, activating oncogenes and suppressor particles in polymerase transcription. Many researchers are seeking new anti-cancer therapies, based on the inhibition of many tumorigenic nucleolar activities, including polymerase activities. Drug candidates, such as CX-3543 and CX-3461, which act as small molecule inhibitors, have been developed targeting RNA polymerase II (6). Many pharmaceutical companies are trying to find therapeutic targets, including nanoparticles (41), for various nucleolar molecules in oncogenesis (40, 42, 43).

## CONCLUSION

Molecular dissection of the composition, assembly, and maintenance of nucleolus components via technological advances is a new research topic in cellular sciences. Coupled with a growing appreciation of the pathological implications of their dysfunction, study of nucleolus components can provide new insights into their role as multifunctional signaling hubs, which play key roles in cellular structures and functions both in health and disease. The activity and contribution of the nucleolus in cancer have been challenging issues for a long time, as they appear to be very complex processes. Until almost the end of the 20^th^ century, morphological changes of nucleoli were only observed in cancer tissues. They were believed to be a consequence of increased metabolic necessities, which characterize proliferating cells. Today, this concept has changed dramatically. Researchers have shown that many gene alterations in the nucleolus (apart from conventional ribosome biogenesis) are responsible for the upregulation of rRNA transcriptional activities in the nucleoli in human tumors. Moreover, new discoveries about the involvement of snoRNAs in many cancers, in particular NSCLC, have become important research topics. It seems that microRNAs act by both initiating cancer cells and inhibiting them for therapeutic purposes.
